# Effect of preoperative pupil offset on corneal higher-order aberrations after femtosecond laser-assisted in situ keratomileusis

**DOI:** 10.1186/s12886-023-02960-y

**Published:** 2023-06-01

**Authors:** Zhanglin Liu, Yang Zhao, Shengshu Sun, Yuan Wu, Guiqin Wang, Shaozhen Zhao, Yue Huang

**Affiliations:** 1 Tianjin Medical University Eye Hospital, Eye Institute and School of Optometry, Tianjin Key Laboratory of Retinal Functions and Diseases, Tianjin Branch of National Clinical Research Center for Ocular Disease, Tianjin, 300384 China; 2 Aier Eye Hospital, Shanxi, 030006 China

**Keywords:** Refractive errors, Femtosecond laser in situ keratomileusis, Pupil offset, Corneal higher-order aberrations

## Abstract

**Background:**

This study aimed to investigate the relationship between multiple higher-order aberrations (HOAs) subgroups and pupil offset, as well as to analyze the factors affecting postoperative corneal HOAs in patients with different degrees of refractive errors.

**Methods:**

We enrolled 160 patients (316 eyes) aged ≥ 18 years who had undergone femtosecond laser-assisted in situ keratomileusis (FS-LASIK) treatment. Based on the relationship between the preoperative pupil offset and the postoperative ΔHOAs, all patients were divided into two groups: group I (pupil offset ≤ 0.20 mm) and group II (pupil offset > 0.20 mm). All of the eyes had low to high myopia with or without astigmatism (manifest refraction spherical equivalent (MRSE) < -10.00 D). Uncorrected distance visual acuity, corrected distance visual acuity, MRSE, pupil offset, central corneal thickness, corneal HOAs, vertical coma (Z_3_^−1^), horizontal coma (Z_3_^1^), spherical aberration (*Z*_4_^0^), trefoil 0° (Z_3_^3^), and trefoil 30° (Z_3_^−3^) over a 6 mm diameter central corneal zone diameter were evaluated preoperatively and at 1 and 3 months postoperatively.

**Results:**

Our result revealed significant differences in postoperative corneal total root mean square (RMS) HOAs, RMS vertical coma, RMS horizontal coma, RMS spherical aberration, and RMS trefoil 30° between group I and group II. ΔMRSE was found to be an effective factor for ΔRMS HOAs (R^2^ = 0.383), ΔRMS horizontal coma (R^2^ = 0.205), and ΔRMS spherical aberration (R^2^ = 0.397). In group II, multiple linear regression analysis revealed a significant correlation between preoperative pupillary offset and Δtotal RMS HOAs (R^2^ = 0.461), ΔRMS horizontal coma (R^2^ = 0.040), and ΔRMS trefoil 30°(R^2^ = 0.089). The ΔRMS vertical coma effect factor is the Y-component, and the factor influencing ΔRMS spherical aberration was ΔMRSE (R^2^ = 0.256).

**Conclusion:**

A small pupil offset was associated with a lower induction of postoperative corneal HOAs. Efforts to optimize centration are critical for improving surgical outcomes in patients with FS-LASIK.

## Background

In correcting hyperopia, myopia, and astigmatism, femtosecond laser in situ keratomileusis (FS-LASIK) has demonstrated excellent safety, efficacy, predictability, and stability and is widely used in clinical procedures [1, 2]. A good outcome of keratomileusis is evaluated by the patient’s vision recovery after surgery and the quality of vision.

One of the most important factors influencing postoperative visual quality is the angle kappa, formed by the pupil axis and visual axis [3–5]. The angle kappa is an important parameter for characterizing the intersection angle of the visual and pupillary axes, which is difficult to measure directly. As the corneal entry point of the visual axis, the corneal coaxial light reflex is closest to it. Furthermore, the corneal coaxial light reflex is the most extensively used method for determining the corneal vertex [6]. Therefore, angle kappa is the distance between the pupil center and the corneal vertex. The angle kappa is related to pupil offset and can be used to cross-reference clinical studies of the anterior ocular segment [7–9]. The ideal center of the cut in the FS-LASIK procedure should be close to the visual axis, but this is difficult to determine intraoperatively, and the active eye tracking system of the excimer laser treatment device is usually positioned to track the pupil center. To the best of our knowledge, studies [10–14] have shown that intraoperative pupillary positioning tracking scans that do not account for angle kappa adjustments can result in “surgically derived” decentration ablation, which can result in increased postoperative higher-order aberrations (HOAs) and reduced visual quality, such as halos, glare, and poor night vision. There have been few studies on the approximate value of the preoperative pupil offset that leads to a significant increase in postoperative HOAs. If the intraoperative decentration ablation from the pupil offset is not taken into account in patients with a large pupillary offset, the reduction in postoperative visual quality may be more pronounced.

Therefore, it is critical to accurately understand the relationship between preoperative pupil offset and HOAs increased postoperatively and adjusted for any pupil offset that may be required to minimize the postoperative increase in HOAs. Therefore, our study investigated the association between multiple HOAs subgroups and pupil offset and analyzed the factors affecting postoperative corneal HOAs in patients with different degrees of refractive error. It can be used as a guide for clinicians who need to adjust the cutting concentration during FS-LASIK.

## Methods

We enrolled 160 Patients (316 eyes) with low to high myopia (with or without astigmatism) at Tianjin Medical University Eye Hospital. The patients were divided into two groups based on the segmental regression equation breakpoints between the preoperative pupil offset and the postoperative HOAs (root mean square (RMS) value of the HOAs.): pupil offset ≤ 0.20 mm and pupil offset > 0.20 mm.

The inclusion criteria were as follows: age patients ≥ 18 years old, refractive error stable for 2 years (annual increase in myopic refractive error not exceeding 0.5 D), soft contact lens wearing discontinued at least 2 weeks before the preoperative examination, and hard contact lens wearing discontinued at least 1 month before the preoperative examination. -0.25D ≤ manifest refractive spherical equivalent, ≤ -10.00D; no keratoconus tendency, active eye diseases, or systemic diseases.

Exclusion criteria included abnormal or keratoconus topography, active ocular inflammation, periocular suppuration, severe ocular appendage lesions, history of previous ocular surgery, concurrent ocular and systemic diseases that may affect corneal wound healing. Written informed consent was obtained from all the patients.

### Preoperative and postoperative eye examinations

Uncorrected distance visual acuity (UDVA), corrected distance visual acuity (CDVA), manifest refraction spherical equivalent (MRSE), slit-lamp assessment of the anterior and posterior segments, and corneal topography were all performed on all patients prior to surgery. An auto kerato-refractometer (KR-800, Topcon, Japan) and an auto chart projector were used to check for refraction. A 3D anterior segment analysis system (Pentacam HR, Oculus, Germany) was used to examine corneal curvature, central corneal thickness (CCT), and corneal HOAs. The patients were positioned and instructed to focus on automatic measurement immediately after blinking to avoid interference caused by the patient’s poor tear film quality and eyelid occlusion. The image quality result was deemed adequate, and the corneal exposed area was > 8 mm. In a diameter range of 6 mm of pupil center, the corneal total HOAs, vertical coma (Z_3_^−1^), horizontal coma (Z_3_^1^), spherical aberration (Z_4_^0^), trefoil 0° (Z_3_^3^), and trefoil 30° (Z_3_^−3^) were measured from the Zernike polynomials analysis. All examinations and data collection were performed under the same conditions by skilled ophthalmologists and operators. Each eye was measured three times, and the mean value of the three measurements was used as the final pupil offset for each patient to ensure the data's reliability. All measurements were performed without pupil dilation, and the ambient lighting conditions were the same throughout the measurements. Before and after surgery, visual acuity, corneal aberrations, corneal curvature, and CCT were assessed and followed up at 1 and 3 months postoperative.

### Surgical procedure

The eyes for FS-LASIK were anesthetized with 0.4% oxybuprocaine hydrochloride eye drops, and the eyelids were opened using an eyelid opener. A negative-pressure suction ring was placed in the center of the cornea after the corneal surface had been smoothed. The eyeball was fixed with negative pressure when the corneoscleral edge and the center of the negative pressure suction ring coincided. The corneal flap was created using IntraLase FS laser equipment (USA). The flap diameter was 8.5 mm, and the hinge was above it. The optical ablation zone was 6.3 mm in diameter. After all, the bubbles under the corneal flap had been absorbed, the corneal flap was split using a splitter, and the stromal bed was cut with a Schwind Amaris 1050RS excimer laser system. The corneal flap was restored after cutting by washing with a balanced salt solution. Both the intraoperative and preoperative lighting conditions were the same.

### Statistical analysis

SPSS statistical software (version 26.0; SPSS, Chicago, Illinois, United States) and GraphPad Prism Software (version 9.0) were used for statistical analysis. Curve estimation and nonlinear regression were expressed as piecewise linear regression. The Kolmogorov–Smirnov test was used to determine whether the parameters had a Gaussian distribution. We used the student’s t-test and Mann–Whitney U test for continuous variables to assess statistical differences. The Spearman rank correlation test was used to examine the relationship between variables, expressed as the Spearman correlation coefficient. We used multinomial linear regression analysis to investigate the factors influencing the increase in HOAs and its subgroups. Statistical significance was defined as a two-tailed *p*-value < 0.05.

## Results

During the observation period, no postoperative complication affecting vision was observed during any surgical procedures.

**Fig. 1:**
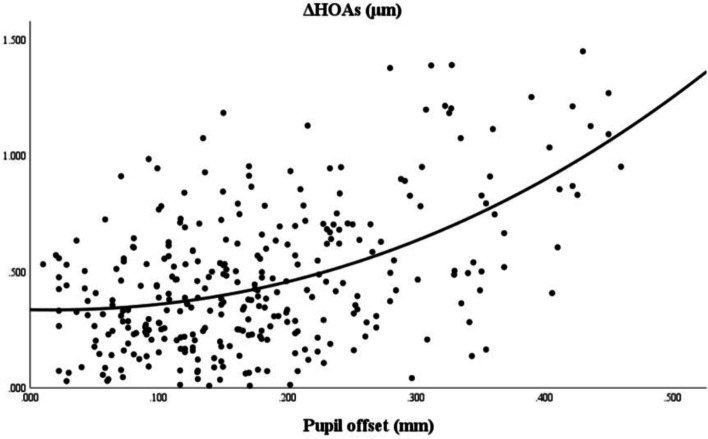
The curve estimation analysis of the association between pupil offset and ΔHOAs

**Fig. 2:**
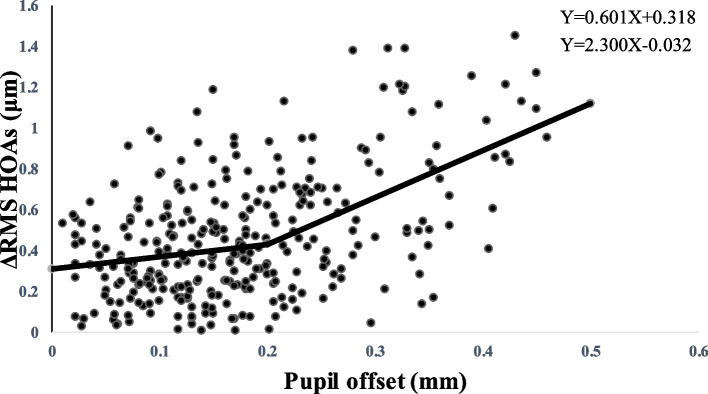
The nonlinear regression analysis of the association between pupil offset and ΔHOAs

The piecewise linear regression analysis (Fig. 1 (The curve estimation analysis of the association between pupil offset and ΔHOAs) and Fig. 2 (The nonlinear regression analysis of the association between pupil offset and ΔHOAs)) revealed that the estimated breakpoint between ΔRMS HOAs and pupillary offset is 0.20 mm. Based on this result, the patients were divided into two groups: group I (pupil offset ≤ 0.20 mm) and group II (pupil offset > 0.20 mm). Table 1 shows demographic data and eye characteristics. The 1-month and 3-month follow-up examinations were attended by all 160 patients (316 eyes). The patients’ mean age was 26.26 ± 5.79, and most were women (91 cases). 0.06 was more common in patients with preoperative UDVA, and 1.2 was more common in patients with postoperative CDVA. Most patients had a median pupil diameter of 2.95 mm. Table 2 shows comparisons of eye characteristics according to pupil offset. Groups I and II included 211 and 105 eyes, respectively. In terms of UDVA, cylinder, pupil diameter, total RMS HOAs, and its subgroups, there were no statistically significant differences between the two groups (*P* > 0.05). There were statistically significant differences between the two groups in spherical, MRSE, CCT, pupil offset, X-component, and Y-component measurements (*P* < 0.05).

**Table 1:** The demographic data and characteristics of eyes

Demographics	
**Cases, eyes, n**	316
**Age, years**^a^	26.26 ± 5.79 (17–49)
**Sex, male/female**	69/91
**UDVA**	0.06 (0.04–0.12)
**CDVA**	1.2 (1.2–1.5)
**Refractive errors, D**^a^	
Spherical	-5.28 ± 1.83 (-9.25 to -0.5)
Cylindrical	-0.75 ± 0.55 (-2.75 to -0.75)
MRSE	-5.65 ± 1.86 (-9.88 to -1.00)
**K1,Flap curvature, D**^a^	42.91 ± 1.40 (39.8–46.9)
**K2,Steep curvature, D**^a^	44.04 ± 1.44 (40.6–47.9)
**Pupil diameter, mm**	2.95 (2.65–3.45)
**CCT, μm**	544 (520–569)
**Preoperative pupillary offset, mm**	
X-component	0.09 (0.04–0.16)
Y-component	0.10 (0.05–0.15)
Pupiloffset	0.16 (0.10–0.23)
**Total RMS HOAs, μm**	0.395 (0.345–0.473)
**RMS Vertical coma, μm**	0.137 (0.07–0.220)
**RMS Horizontal coma, μm**	0.088 (0.039–0.135)
**RMS Spherical aberration, μm**	0.204 (0.159–0.256)
**RMS Trefoil 0°, μm**	0.072 (0.030–0.124)
**RMS Trefoil 30°, μm**	0.099 (0.047–0.163)

Results are expressed as Median (P25-P75), ^a^Means ± standard deviation (range), *D* Diopters, *CCT* Central corneal thickness. *MRSE* Manifest refraction spherical equivalent, *HOAs* Higher-order aberrations (3rd to 7th order), *RMS* Root mean square. *UDVA* Uncorrected distance visual acuity, *CDVA* Corrected distance visual acuity

**Table 2:** Comparisons for characteristics in eyes according to degree of pupil offset

Variables	Group I (Pupil offset ≤ 0.20 mm)	Group II (Pupil offset > 0.20 mm)	*P*
**Cases, eyes, n**	211	105	
**UDVA**	0.06 (0.04–0.12)	0.05 (0.04–0.12)	0.133
**Refractive errors, D**^a^			
Spherical	-5.02 ± 1.86 (-9.25 to -0.5)	-5.78 ± 1.67 (-9.25 to -0.75)	**0.000**
Cylindrical	-0.75 ± 0.53 (-2.25 to -0.75)	-0.81 ± 0.56 (-2.75 to 0.00)	0.152
MRSE	-5.38 ± 1.87 (-9.63 to -1.00)	-6.19 ± 1.73 (-9.88 to -1.25)	**0.000**
**K1,Flap curvature, D**^a^	43.01 ± 1.43 (39.8–46.9)	42.72 ± 1.34 (40.5–46.7)	0.087
**K2,Steep curvature, D**^a^	44.11 ± 1.50 (40.6–47.7)	43.89 ± 1.32 (41.6–47.9)	0.199
**Pupil diameter, mm**	2.94 (2.65–3.46)	2.95 (2.56–3.42)	0.378
**CCT, μm**	540 (517–564)	551 (531–577)	**0.001**
**Preoperative pupillary offset, mm**			
X-component	0.06 (0.03–0.11)	0.18 (0.11–0.24)	**0.000**
Y-component	0.07 (0.04–0.12)	0.19 (0.10–0.27)	**0.000**
Pupiloffset	0.12 (0.08–0.16)	0.27 (0.23–0.34)	**0.000**
**Total RMS HOAs, μm**	0.399 (0.353–0.471)	0.385 (0.325–0.474)	0.224
**RMS Vertical coma, μm**	0.136 (0.078–0.219)	0.137 (0.065–0.223)	0.652
**RMS Horizontal coma, μm**	0.092 (0.039–0.140)	0.079 (0.028–0.129)	0.199
**RMS Spherical aberration, μm**	0.209 (0.167–0.256)	0.186 (0.148–0.253)	0.192
**RMS Trefoil 0°, μm**	0.067 (0.029–0.124)	0.074 (0.034–0.125)	0.434
**RMS Trefoil 30°, μm**	0.108 (0.051–0.170)	0.092 (0.044–0.158)	0.301

Results are expressed as Median (P25-P75); ^a^Means ± standard deviation (range); *D* Diopters, *CCT* Central corneal thickness. *MRSE* Manifest refraction spherical equivalent, *HOAs* Higher-order aberrations (3rd to 7th order), *RMS* Root mean square. Significant values are shown in bold. *P*-values < 0.05 were considered significant; *UDVA* Uncorrected distance visual acuity

**Fig. 3:**
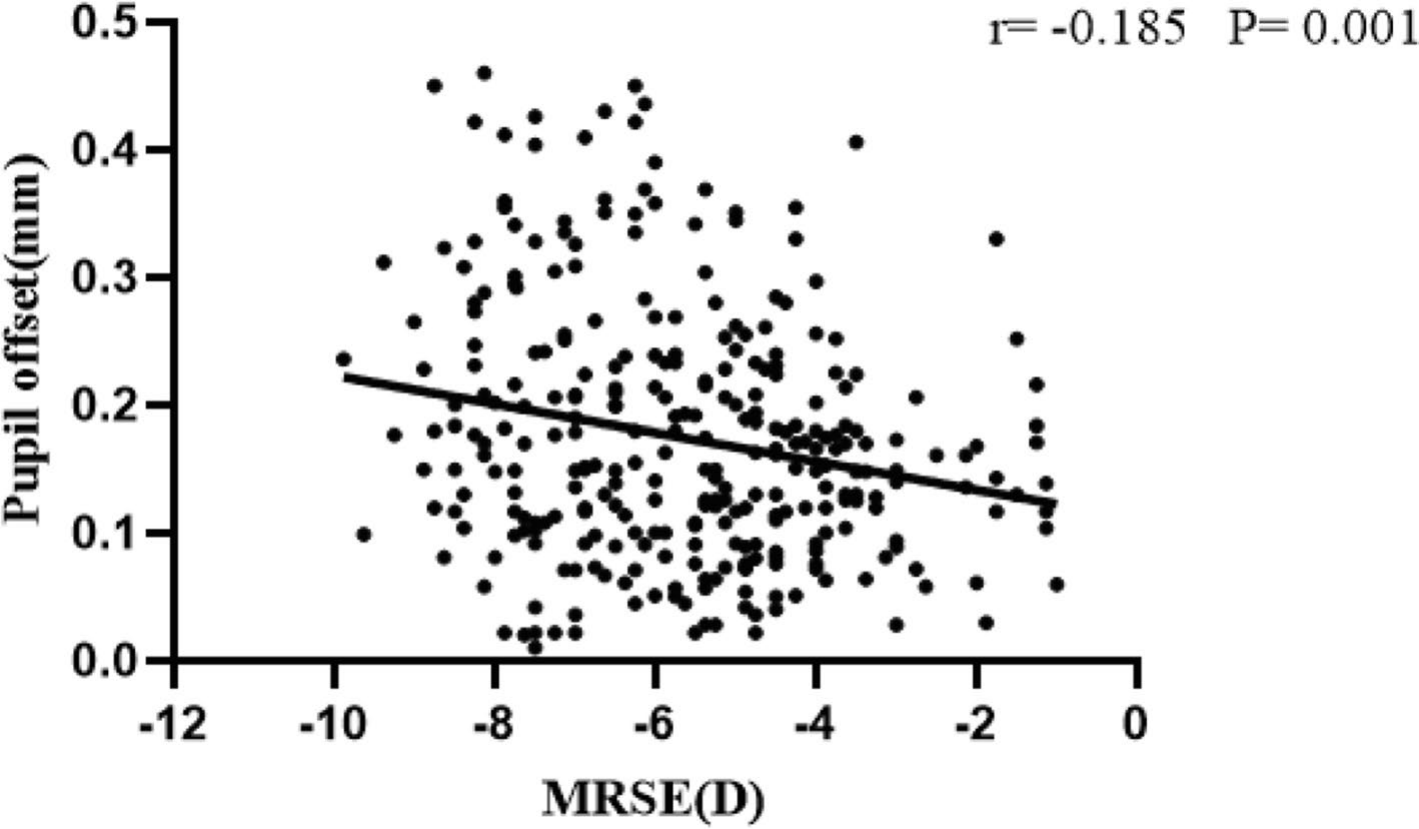
The correlation analysis between MRSE and pupil offset in all patients

**Fig. 4:**
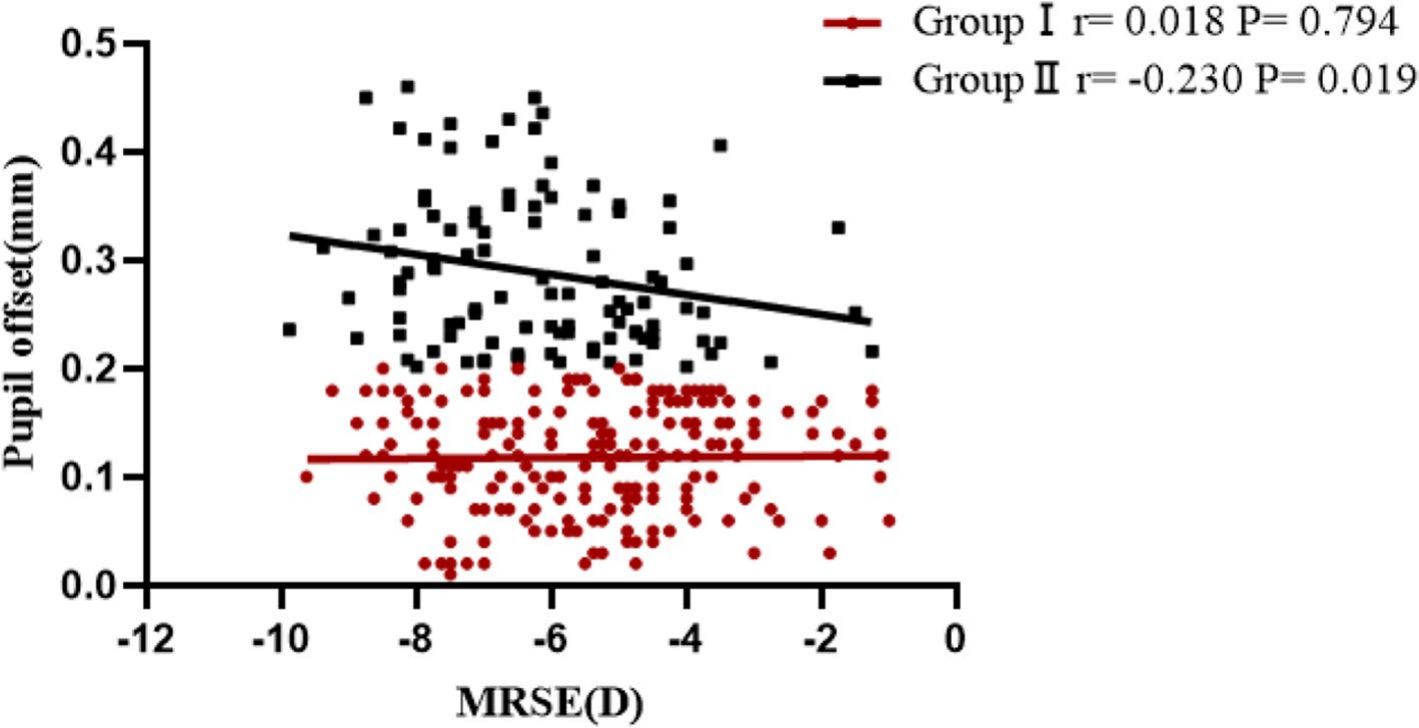
The correlation analysis between MRSE and pupil offset according to the degree of pupil offset. Group I, pupil offset ≤ 0.20 mm; Group II > 0.20 mm

Figure 3 (The correlation analysis between MRSE and pupil offset in all patients) shows that the preoperative pupil offset was correlated with MRSE (*r* = -0.185, *P* = 0.001). Figure 4 (The correlation analysis between MRSE and pupil offset according to the degree of pupil offset) shows that the preoperative pupil offset was not correlated with MRSE in group I (*r* = 0.018, *P* = 0.794), but was correlated with MRSE in group II (*r* = -0.230, *P* = 0.019).

**Fig. 5:**
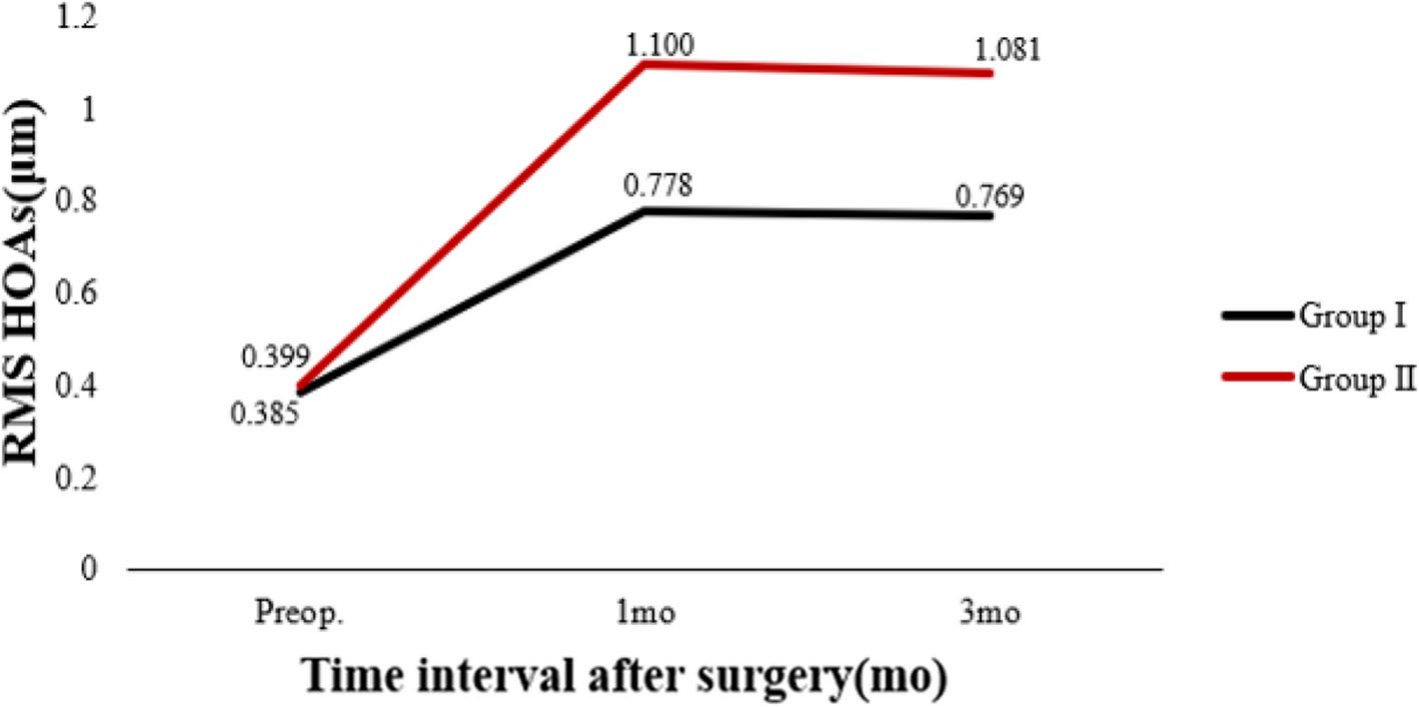
The preoperative and postoperative changes of corneal RMS HOAs and its subgroups. Group I: pupil offset ≤ 0.20 mm; Group II: pupil offset > 0.20 mm

**Fig. 6:**
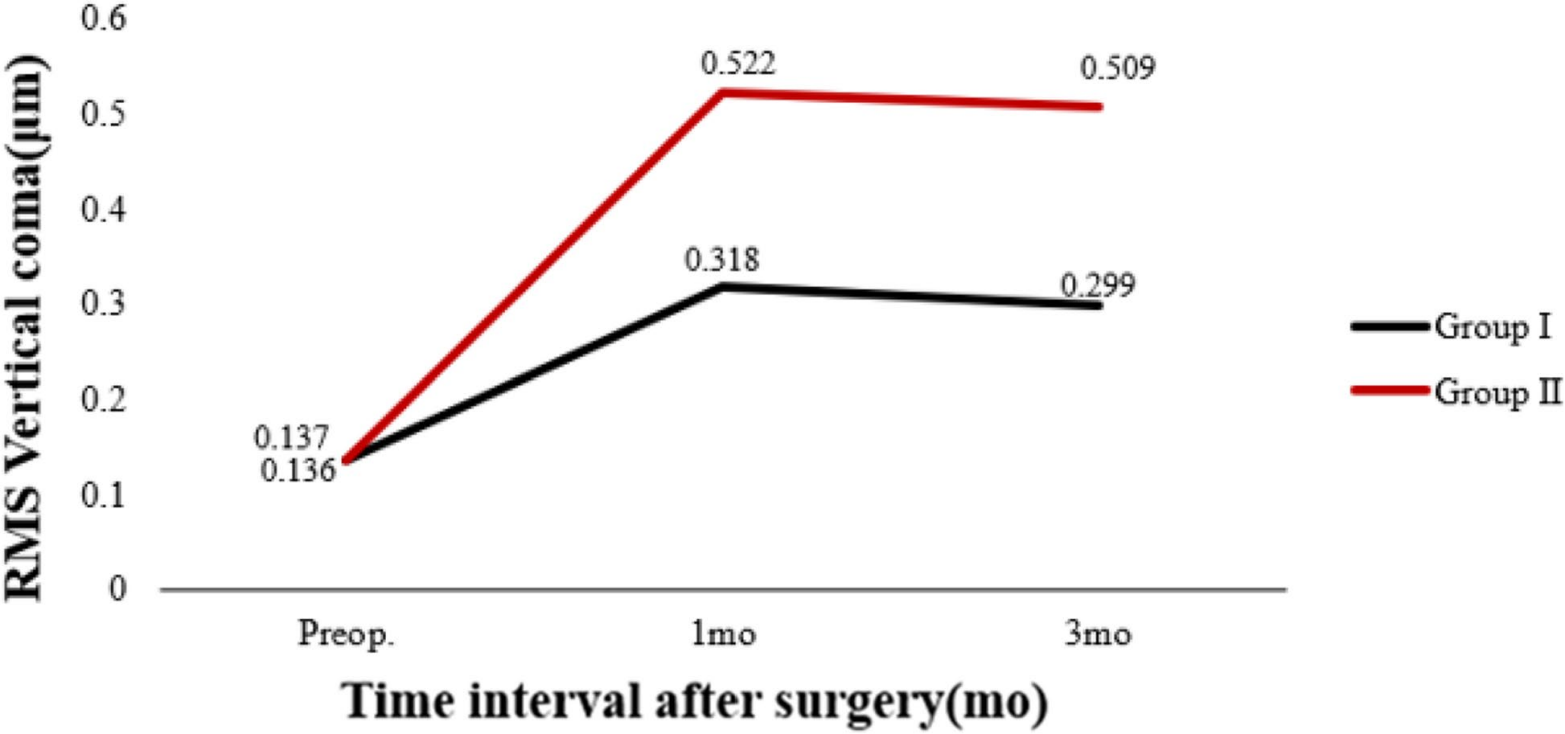
The preoperative and postoperative changes of corneal RMS HOAs and its subgroups. Group I: pupil offset ≤ 0.20 mm; Group II: pupil offset > 0.20 mm

**Fig. 7:**
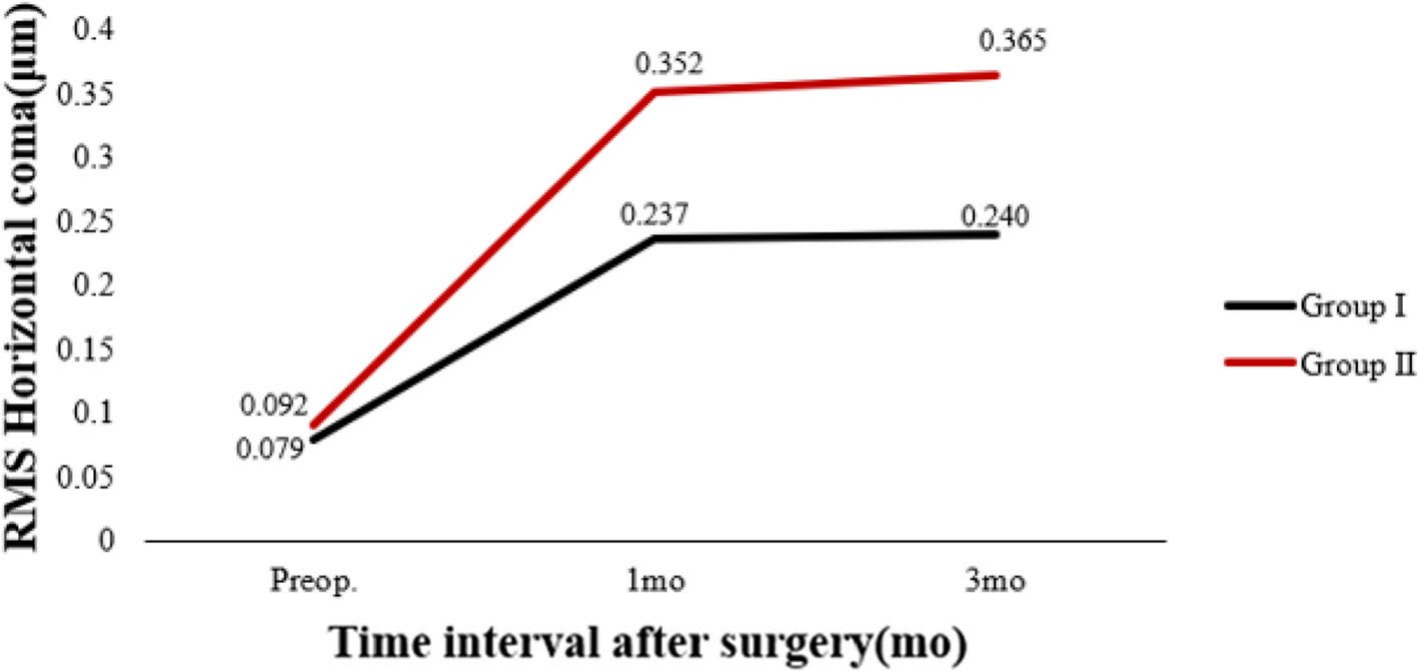
The preoperative and postoperative changes of corneal RMS HOAs and its subgroups. Group I: pupil offset ≤ 0.20 mm; Group II: pupil offset > 0.20 mm

**Fig. 8:**
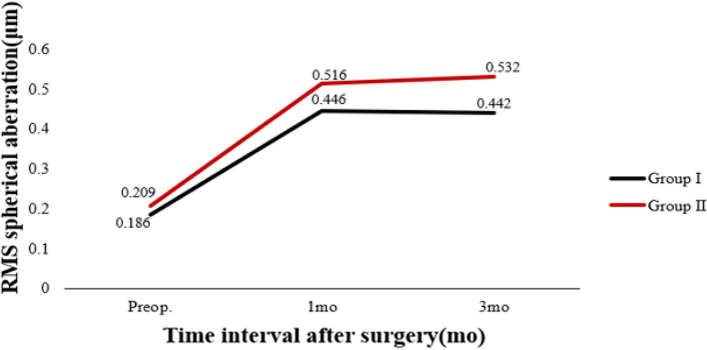
The preoperative and postoperative changes of corneal RMS HOAs and its subgroups. Group I: pupil offset ≤ 0.20 mm; Group II: pupil offset > 0.20 mm

**Fig. 9:**
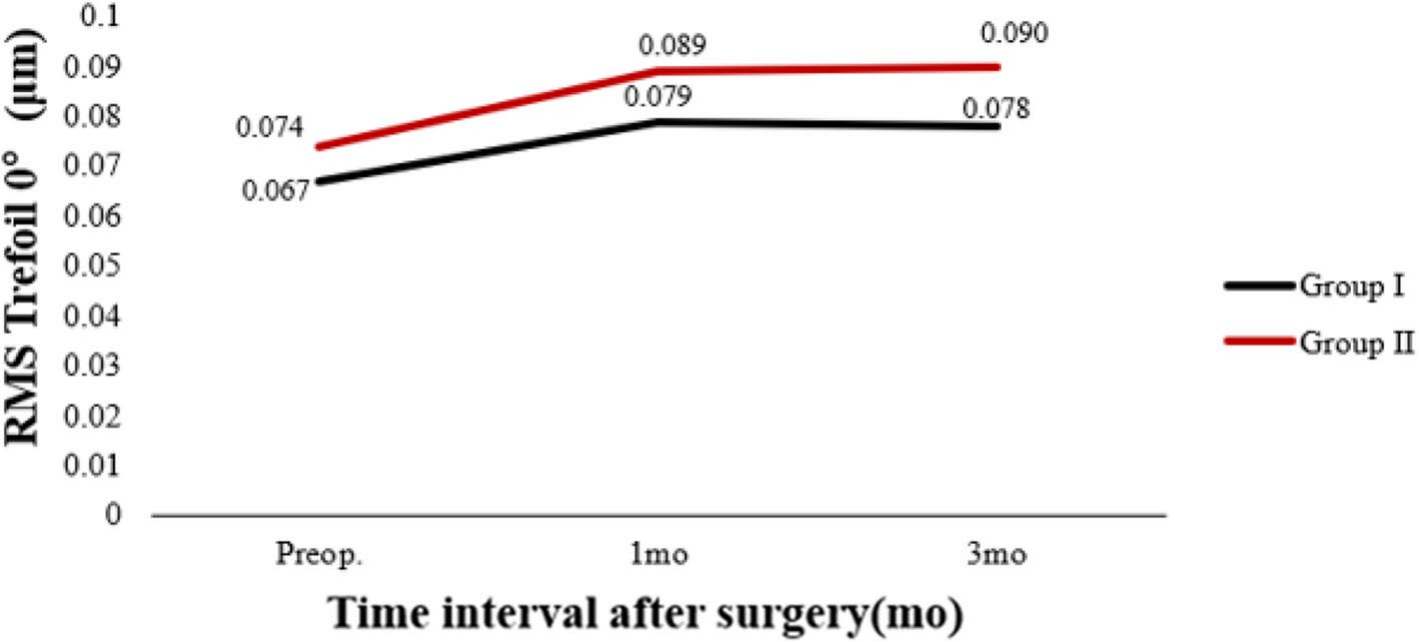
The preoperative and postoperative changes of RMS HOAs subgroups, CCT and Visual acuity. Group I: pupil offset ≤ 0.20 mm; Group II: pupil offset > 0.20 mm

**Fig. 10:**
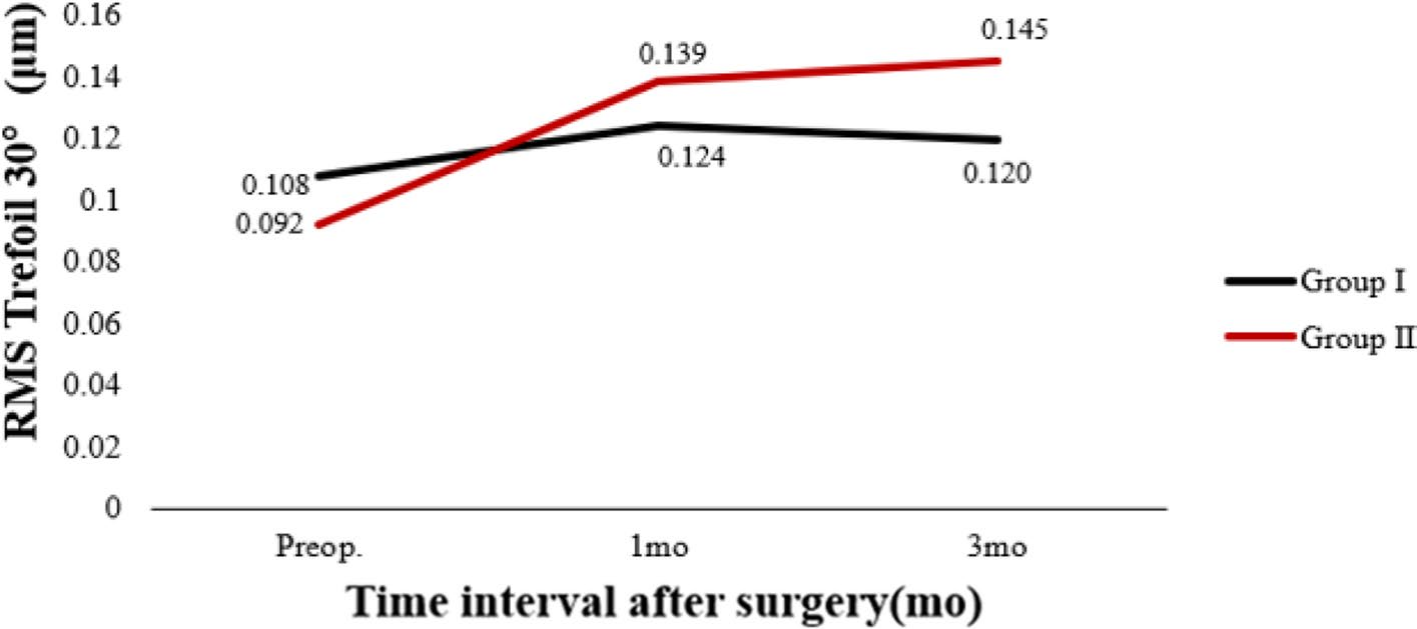
The preoperative and postoperative changes of RMS HOAs subgroups, CCT and Visual acuity. Group I: pupil offset ≤ 0.20 mm; Group II: pupil offset > 0.20 mm

**Fig. 11:**
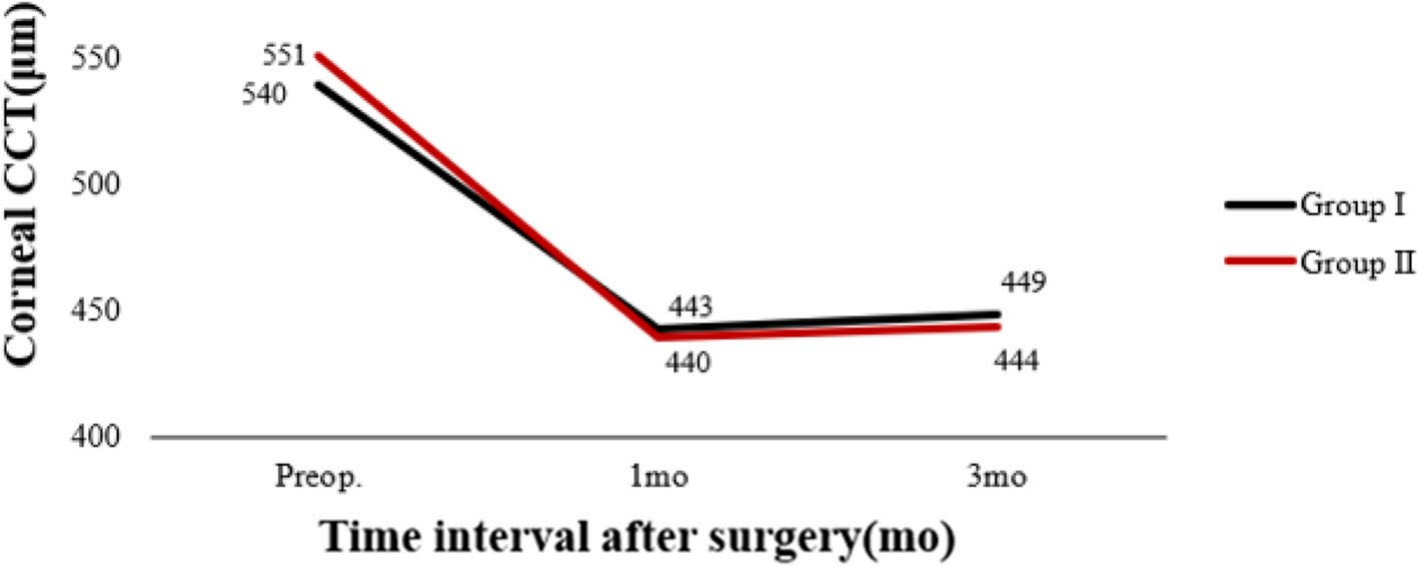
The preoperative and postoperative changes of RMS HOAs subgroups, CCT and Visual acuity. Group I: pupil offset ≤ 0.20 mm; Group II: pupil offset > 0.20 mm

**Fig. 12:**
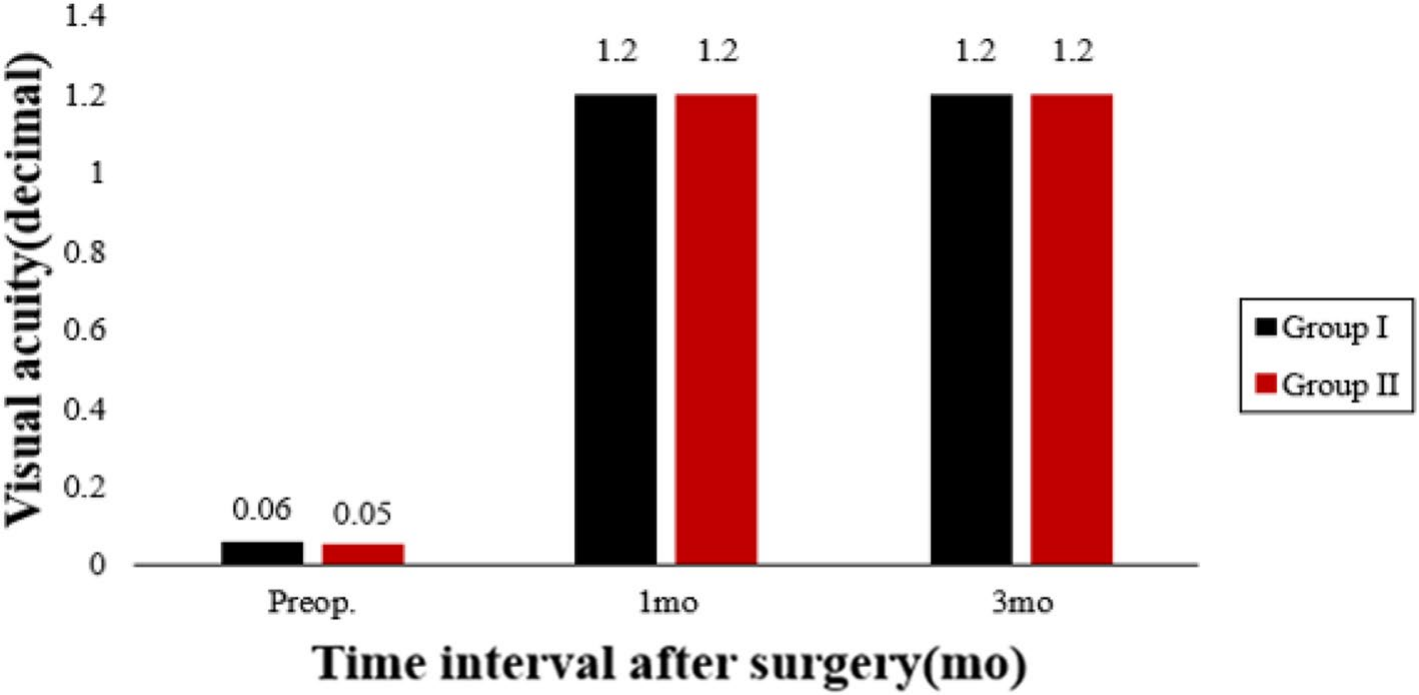
The preoperative and postoperative changes of RMS HOAs subgroups, CCT and Visual acuity. Group I: pupil offset ≤ 0.20 mm; Group II: pupil offset > 0.20 mm

Table 3 shows preoperative and postoperative analyses of ΔHOAs and ΔCCT in eyes that underwent FS-LASIK. In terms of preoperative CCT, there were statistically significant differences between the two groups. In both groups, there were statistically significant differences 3 months postoperatively and preoperatively. There was a significant difference in ΔCCT between group I and group II (*P* < 0.05). The preoperative RMS HOAs and its subgroups did not differ significantly between groups I and II (P > 0.05). RMS HOAs increased in both groups 3 months postoperatively compared to preoperatively. The ΔRMS HOAs, ΔRMS vertical coma, ΔRMS horizontal coma, ΔRMS spherical aberration, and ΔRMS trefoil 30 were all higher in group II than in group I (*P* < 0.05). Figures 5, 6, 7, 8, 9, 10, 11 (Time interval after surgery) shows the changes in corneal CCT, RMS HOAs, and its subgroups 1 and 3 months after surgery. Figure 12 (Time interval after surgery) shows preoperative and postoperative 1 and 3 months visual acuity.

**Table 3:** Analysis of corneal HOAs and CCT in eyes that underwent FS-Lasik

**Groups**	**Preop.**	**3mo**	**Δ**	***P ***(preop. vs 3mo)
**CCT, μm**				
Group I	540 (517–564)	449 (416–481)	91 (72–118)	**0.000**
Group II	551 (531–577)	444 (412–477)	110 (85–132)	**0.000**
***P***	**0.001**	0.331	**0.000**	
**Total RMS HOAs, μm**				
Group I	0.399 (0.353–0.471)	0.769 (0.634–0.916)	0.346 (0.211–0.528)	**0.000**
Group II	0.385 (0.325–0.474)	1.081 (0.928–1.388)	0.720 (0.460–0.962)	**0.000**
***P***	0.224	**0.000**	**0.000**	
**RMS Vertical coma, μm**				
Group I	0.136 (0.078–0.219)	0.299 (0.179–0.462)	0.153 (0.020–0.282)	**0.000**
Group II	0.137 (0.065–0.223)	0.509 (0.240–0.780)	0.304 (0.131–0.637)	**0.000**
***P***	0.652	**0.000**	**0.000**	
**RMS Horizontal coma, μm**				
Group I	0.092 (0.039–0.140)	0.240 (0.140–0.406)	0.140 (0.036–0.284)	**0.000**
Group II	0.079 (0.028–0.129)	0.365 (0.143–0.592)	0.272 (0.086–0.508)	**0.000**
***P***	0.199	**0.004**	**0.000**	
**RMS Spherical aberration, μm**				
Group I	0.209 (0.167–0.256)	0.442 (0.336–0.545)	0.235 (0.124–0.338)	**0.000**
Group II	0.186 (0.148–0.253)	0.532 (0.420–0.636)	0.323 (0.220–0.439)	**0.000**
***P***	0.192	**0.000**	**0.000**	
**RMS Trefoil 0°, μm**				
Group I	0.067 (0.029–0.124)	0.078 (0.033–0.149)	0.013 (-0.035–0.060)	0.155
Group II	0.074 (0.034–0.125)	0.090 (0.036–0.179)	0.008 (-0.046–0.084)	0.355
***P***	0.434	0.540	0.868	
**RMS Trefoil 30°, μm**				
Group I	0.108 (0.051–0.170)	0.120 (0.066–0.194)	0.020 (-0.058–0.089)	**0.034**
Group II	0.092 (0.044–0.158)	0.145 (0.085–0.240)	0.062 (-0.021–0.155)	**0.000**
***P***	0.301	**0.023**	**0.005**	

Results are expressed as Median (P25-P75); *HOAs* Higher-order aberrations (3rd to 7th order), *RMS* Root mean square. Significant values are shown in bold. *P*-values < 0.05 were considered significant

Table 4 shows that MRSE was correlated with Δtotal RMS HOAs (*r* = -0.614, *P* < 0.001**),** ΔRMS vertical coma (*r* = -0.156, *P* = 0.024), ΔRMS horizontal coma (*r* = -0.410, *P* < 0.001), ΔRMS spherical aberration (*r* = -0.592, *P* < 0.001), and ΔRMS trefoil 30° (*r* = -0.147, *P* = 0.033). ΔCCT was correlated with Δtotal RMS HOAs (*r* = 0.511, *P* < 0.001)**,** ΔRMS vertical coma (*r* = 0.177, *P* < 0.001), ΔRMS horizontal coma (*r* = 0.326, *P* < 0.001), and ΔRMS spherical aberration (*r* = 0.415, *P* < 0.001). Pupil offset was correlated with ΔRMS vertical coma (*r* = 0.186, *P* = 0.007). The Y-component was correlated with ΔRMS vertical coma (*r* = 0.222, *P* = 0.001).

**Table 4:** Correlation of corneal parameters and induced corneal HOAs after FS-Lasik in Group I (Pupil offset ≤ 0.20 mm)

Parameters	Δ Total RMS HOAs		Δ RMS Vertical coma		Δ RMS Horizontal coma		Δ RMS Spherical aberration		Δ RMS Trefoil 0°		Δ RMS Trefoil 30°	
	r	***P***	r	***P***	r	***P***	r	***P***	r	***P***	r	***P***
**ΔRefractive errors, D**												
Spherical	-0.627	**0.000**	-0.152	**0.028**	-0.436	**0.000**	-0.610	**0.000**	0.060	0.384	-0.143	**0.038**
Cylindrical	0.036	0.601	-0.028	0.690	0.137	**0.047**	0.070	0.308	-0.058	0.405	-0.024	0.727
MRSE	-0.614	**0.000**	-0.156	**0.024**	-0.410	**0.000**	-0.592	**0.000**	0.055	0.426	-0.147	**0.033**
**ΔCCT, μm**	0.511	**0.000**	0.177	**0.000**	0.326	**0.000**	0.415	**0.000**	-0.054	0.433	0.085	0.217
**Preoperative pupillary offset, mm**												
X-component	0.029	0.673	-0.017	0.801	0.042	0.547	-0.082	0.235	0.008	0.905	0.086	0.213
Y-component	0.126	0.618	0.222	**0.001**	0.054	0.438	0.079	0.253	-0.091	0.189	0.036	0.601
Pupiloffset	0.126	0.067	0.186	**0.007**	0.085	0.222	-0.018	0.795	-0.033	0.635	0.061	0.378

*MRSE* Manifest refraction spherical equivalent, *HOAs* Higher-order aberrations (3rd to 7th order), *RMS* Root mean square, *CCT* Central corneal thickness. Significant values are shown in bold. *P*-values < 0.05 were considered significant

Table 5 shows that MRSE was correlated with Δtotal RMS HOAs (*r* = -0.629, *P* < 0.001)**,** ΔRMS vertical coma (*r* = -0.367, *P* < 0.001**),** ΔRMS horizontal coma (*r* = -0.380, *P* < 0.001), ΔRMS spherical aberration (*r* = -0.533, *P* < 0.001**),** and ΔRMS trefoil 30° (*r* = -0.211, *P* = 0.031). ΔCCT was correlated with Δtotal RMS HOAs (*r* = 0.541, *P* < 0.001), ΔRMS vertical coma (*r* = 0.319, *P* < 0.001), ΔRMS horizontal coma (*r* = 0.287, *P* < 0.001), and ΔRMS spherical aberration (*r* = 0.487, *P* < 0.001**).** Pupil offset was correlated with ΔRMS HOAs (*r* = 0.412, *P* < 0.001), ΔRMS horizontal coma (*r* = 0.249, *P* = 0.010), and ΔRMS trefoil 30°(*r* = 0.247, *P* = 0.010); the X-component was correlated with ΔRMS vertical coma (*r* = -0.447, *P* < 0.001) and ΔRMS horizontal coma (*r* = 0.266, *P* = 0.006); and the Y-component was correlated with ΔRMS HOAs (*r* = 0.390, *P* < 0.001) and ΔRMS vertical coma (*r* = -0.472, *P* < 0.001).

**Table 5:** Correlation of corneal parameters and induced corneal HOAs after FS-Lasik in Group II (Pupil offset > 0.20 mm)

Parameters	Δ Total RMS HOAs		Δ RMS Vertical coma		Δ RMS Horizontal coma		Δ RMS Spherical aberration		Δ RMS Trefoil 0°		Δ RMS Trefoil 30°	
	r	***P***	r	***P***	r	***P***	r	***P***	r	***P***	r	***P***
**ΔRefractive errors, D**												
Spherical	-0.626	**0.000**	-0.354	**0.000**	-0.386	**0.000**	-0.520	**0.000**	-0.036	0.715	-0.206	**0.035**
Cylindrical	-0.164	0.094	-0.131	0.183	-0.034	0.727	-0.113	0.249	-0.180	0.066	-0.057	0.564
MRSE	-0.629	**0.000**	-0.367	**0.000**	-0.380	**0.000**	-0.533	**0.000**	-0.062	0.533	-0.211	**0.031**
**ΔCCT, μm**	0.541	**0.000**	0.319	**0.000**	0.287	**0.000**	0.487	**0.000**	0.083	0.397	0.167	0.088
**Preoperative pupillary offset, mm**												
X-component	-0.177	0.072	-0.447	**0.000**	0.266	**0.006**	-0.050	0.616	-0.072	0.466	-0.118	0.232
Y-component	0.390	**0.000**	-0.472	**0.000**	-0.064	0.514	0.141	0.150	0.168	0.087	0.162	0.099
Pupiloffset	0.412	**0.000**	0.166	0.091	0.249	**0.010**	0.190	0.053	0.064	0.514	0.247	**0.011**

*MRSE* Manifest refraction spherical equivalent, *HOAs* Higher-order aberrations (3rd to 7th order), *RMS* Root mean square, *CCT* Central corneal thickness. Significant values are shown in bold. *P*-values < 0.05 were considered significant

Table 6 shows the results. Multivariate analyses using multiple linear regression revealed that ΔMRSE was the effect factor for ΔRMS HOAs (R^2^ = 0.383, *P* < 0.001), ΔRMS horizontal coma (R^2^ = 0.205, *P* < 0.001), and ΔRMS spherical aberration (R^2^ = 0.397, *P* < 0.001), after adjusting for independent variables correlated with ΔRMS HOAs and its subgroups. The effect factor for ΔRMS horizontal coma was Δcylindrical, and the effect factor for ΔRMS spherical aberration was ΔCCT.

**Table 6:** Multiple linear regression analysis of association between corneal parameters and ΔHOAs after FS-Lasik in Group I (Pupil offset ≤ 0.20 mm)

Parameters	B	Beta	SE	t	*P*
**Adjusted R**^**2**^** = 0.383**					
ΔMRSE on **Δ Total RMS HOAs**	-0.091	-0.739	0.014	-6.355	**0.000**
ΔCCT on **Δ Total RMS HOAs**	-0.001	-0.134	0.001	-1.156	0.249
**Adjusted R**^**2**^** = 0.067**					
ΔMRSE on **Δ RMS Vertical coma**	-0.010	-0.076	0.018	-0.531	0.596
ΔCCT on **Δ RMS Vertical coma**	0.001	0.112	0.001	0.777	0.438
Pupil offset on **Δ RMS Vertical coma**	0.319	0.065	0.413	0.772	0.441
Y-component on **Δ RMS Vertical coma**	0.808	0.164	0.415	1.946	0.053
**Adjusted R**^**2**^** = 0.205**					
ΔCylindrical on **Δ RMS Horizontal coma**	0.078	0.201	0.026	3.059	**0.003**
ΔMRSE on **Δ RMS Horizontal coma**	-0.057	-0.507	0.015	-3.720	**0.000**
ΔCCT on **Δ RMS Horizontal coma**	-0.001	-0.082	0.001	-0.587	0.558
**Adjusted R**^**2**^** = 0.397**					
ΔMRSE on **Δ RMS Spherical aberration**	-0.074	-0.903	0.009	-7.851	**0.000**
ΔCCT on **Δ RMS Spherical aberration**	-0.002	-0.324	0.001	-2.816	**0.005**
**Adjusted R**^**2**^** = 0.008**					
ΔMRSE on **Δ RMS Trefoil 30°**	-0.007	-0.111	0.004	-1.614	0.108

*MRSE* Manifest refraction spherical equivalent, *HOAs* Higher-order aberrations (3rd to 7th order), *RMS* Root mean square, *CCT* Central corneal thickness. Significant values are shown in bold. *P*-values < 0.05 were considered significant

Table 7 shows the results. A significant relationship was found between the preoperative pupil offset and Δtotal RMS HOAs (R^2^ = 0.461, *P* = 0.001), ΔRMS horizontal coma (R^2^ = 0.040, *P* = 0.007), and ΔRMS trefoil 30° (R^2^ = 0.089, *P* = 0.009). The effect factors for the ΔRMS vertical coma and X and Y components. ΔMRSE was the effect factor for ΔRMS spherical aberration (R^2^ = 0.256, *P* = 0.003). There was no multicollinearity between the independent variables in the sample data. The residuals are distributed normally.

**Table 7:** Multiple linear regression analysis of association between corneal parameters and ΔHOAs after FS-Lasik in Group II (Pupil offset > 0.20 mm)

Parameters	B	Beta	SE	t	*P*
**Adjusted R**^**2**^** = 0.461**					
ΔMRSE on **Δ Total RMS HOAs**	-0.103	-0.507	0.024	-4.354	**0.000**
Pupil offset on **Δ Total RMS HOAs**	1.456	0.291	0.414	3.514	**0.001**
ΔCCT on **Δ Total RMS HOAs**	0.000	-0.013	0.001	-0.117	0.907
Y-component on **Δ Total RMS HOAs**	0.389	0.116	0.286	1.359	0.177
**Adjusted R**^**2**^** = 0.304**					
X-component on **Δ RMS Vertical coma**	0.979	0.285	0.346	2.827	**0.006**
Y-component on **Δ RMS Vertical coma**	-1.009	-0.254	0.383	-2.634	**0.010**
ΔMRSE on **Δ RMS Vertical coma**	-0.046	-0.220	0.028	-1.663	0.099
ΔCCT on **Δ RMS Vertical coma**	0.081	0.001	0.001	0.005	0.996
**Adjusted R**^**2**^** = 0.040**					
Pupil offset on **Δ RMS Horizontal coma**	-0.597	-0.281	0.215	-2.776	**0.007**
ΔMRSE on **Δ RMS Horizontal coma**	-0.013	-0.147	0.013	-0.951	0.344
ΔCCT on **Δ RMS Horizontal coma**	-1.219	-0.003	0.001	-0.019	0.985
X-component on **Δ RMS Horizontal coma**	0.097	0.059	0.164	0.594	0.554
**Adjusted R**^**2**^** = 0.256**					
ΔMRSE on **Δ RMS Spherical aberration**	-0.039	-0.408	0.013	-3.033	**0.003**
ΔCCT on **Δ RMS Spherical aberration**	0.001	0.135	0.001	1.001	0.319
**Adjusted R**^**2**^** = 0.089**					
Pupil offset on **Δ RMS Trefoil 30°**	0.567	0.256	0.213	2.660	**0.009**
ΔMRSE on **Δ RMS Trefoil 30°**	-0.014	-0.152	0.009	-1.584	0.949

*MRSE* Manifest refraction spherical equivalent, *HOAs* Higher-order aberrations (3rd to 7th order), *RMS* Root mean square, *CCT* Central corneal thickness. Significant values are shown in bold. *P*-values < 0.05 were considered significant

## Discussion

Doctors are currently focusing on visual quality after refractive surgery. Subjective and objective evaluations are two commonly used methods for assessing visual quality. The former, however, is subject to some subjectivity due to the patient’s cognitive understanding ability and level of cooperation, and the evaluation accuracy and repeatability errors are relatively large. Objective visual quality assessment reduces error due to patient cooperation, has some operability and repeatability, and has a wide range of clinical application values. The pupil diameter was approximately 2.95 mm. If the pupil diameter was 3.00 mm, it has previously been calculated that a decentration of not > 0.20 mm would be required to maintain optical quality [15]. This result, presented in piecewise linear regression, was generally consistent with ours.

We have compared the basic parameters of the two study groups. The difference of MRSE could be explained by the positive correlation between pupil offset and refractive errors. The larger the MRSE, the more obvious pupil offset. In this case, more attention should be paid attention to in preoperative examination. In addition, the difference explained that the greater the preoperative MRSE and pupil offset, the greater the increase of corneal spherical aberration after refractive surgery. On the other hand, there was no significant statistical difference between the total HOAs and its subgroups between the two groups before refractive surgery, but a significant statistical difference after surgery was observed, which makes clearer changes in the relationship between HOAs and its subgroups before and after the surgery, and therefore a more significant conclusion.

Our results provided insight into the association between pupil offset and ΔRMS HOAs as well as its subgroups. Patients with pupil offset > 0.20 mm had a higher postoperative corneal total RMS HOAs and ΔRMS HOAs than those with pupil offset ≤ 0.20 mm. In our study of the relationship between pupil offset and ΔRMS HOAs, we found that in patients with pupil offset > 0.20 mm, pupil offset was associated with ΔRMS HOAs, vertical coma, horizontal coma, and trefoil of 30°. The Y-component correlated with ΔRMS vertical coma. In patients with pupil offsets ≤ 0.20 mm, there were no statistically significant differences in the association between pupil offset and ΔRMS HOAs. This is because the visual axis of the eyes is closer to the pupil axis in patients with a small pupil offset, avoiding intraoperative decentration ablation and induction of more HOAs. The main factor affecting postoperative HOAs was ΔMRSE in eyes with pupil offset ≤ 0.20 mm, consistent with previous studies [16, 17]. According to studies [18–20], decentration from the center of the entrance pupil was associated with greater induction of total RMS HOA, coma, and spherical aberration after surgery. However, the breakpoint between the ΔRMS HOAs and the pupil is affected by different variables in different studies, such as pupil size and refractive errors, among others.

We found that pupil offset was linearly and positively correlated with preoperative MRSE in this study, which was consistent with a previous study [21]. Furthermore, If the pupil offset was > 0.20 mm, there was a statistically significant correlation between pupil offset and MRSE. These results explain the difference in MRSE between groups I and II. We found that ΔCCT was correlated with ΔRMS HOAs, ΔRMS vertical coma, ΔRMS horizontal coma, and ΔRMS spherical aberration in group I and II. However, In Group I, ΔCCT were only correlated with ΔRMS spherical aberration in multiple linear regression. This suggests that the Scheimpflug camera may introduce measurement errors associated with the accuracy and repeatability of corneal thickness in the central zone. Corneal thickness difference maps may not be accurate because the eye’s anterior surface has been significantly altered following refractive surgery [22–24].

During 1 and 3 month follow-up after refractive surgery, we found that changes in patients’ CDVA, CCT, and HOAs tended to be stable at 3 months. Therefore, investigated changes in eye parameters over a 3 month postoperative period. Except for RMS trefoil 0°, a significant difference was found between postoperative RMS HOAs and preoperative RMS HOAs. The ΔRMS HOAs and its subgroups, except for RMS trefoil 0° between groups I and II, were found to be significantly different, especially the RMS vertical coma and RMS spherical aberration. According to Zheng et al.[25], significant HOAs induction is common after refractive surgery, and the formation of a corneal flap, changes in corneal biomechanical properties after surgery, and corneal wound healing may be the causes of the increase [26, 27]. Our results showed that after FS-LASIK, corneal RMS HOAs, RMS corneal coma, RMS spherical aberration, and RMS trefoil 30° were significantly increased indicated that while the surgery eliminated low-order aberrations such as myopia and astigmatism and improved UCVA, it increased corneal RMS HOAs. The rise in vertical coma is thought to be caused by the formation of a corneal flap and minor intraoperative eye movements. The corneal flap is incised for FS-LASIK and the flap hinge is located at the 12 o’clock position of the cornea. Retraction and tension along the hinge axis caused by corneal flap hydration increase asymmetry of the corneal flap relative to the hinge axis, resulting in a change in coma [28]. In addition, the RMS HOAs changes in group II, were greater than those in group I. In group II, a larger pupil offset resulted in a greater increase in RMS vertical coma and RMS spherical aberration. Previous studies have linked coma size to the degree of decentration [29, 30]. Despite the use of a 7-dimensional eye-tracking system in both procedures, the increase in coma caused by eccentric ablation could not be completely avoided due to the uncontrollable factors of patients during the operation.

Our study showed a statistically significant difference in RMS spherical aberration in both groups between the preoperative and postoperative periods. Changes in corneal asphericity caused by corneal epithelial healing and matrix fibrosis are primarily responsible for increased spherical aberration [31, 32]. Furthermore, corneal asphericity was significantly correlated with the preoperative MRSE [25, 33]. Highly myopic patients required more corneal stroma ablation than mild to moderate patients, resulting in significant central corneal thinning, decreased tension, and increased peripheral tension in the central corneal zone. Changes in biomechanical strength may explain why patients with high myopia have more corneal asphericity deformation than those with mild to moderate myopia [34]. The higher the degree of myopia, the deeper the corneal stroma needs to be ablated and the steeper the transition from the center of the cornea to the periphery, the greater the change in asphericity of the cornea, increasing in corneal HOAs, especially spherical aberration in group II.

Several factors were associated with postoperatively induced HOAs. First, aberrations in human eyes were dynamic, with clinical aberrations varying depending on the measurement. The angle kappa and pupil offset were not fixed values [35, 36], but could change depending on the circumstances, such as intraoperative lighting and emotional tension. Therefore, maintaining the same light intensity preoperative and intraoperative is critical. Second, irregularities in the ablation area can affect optical and functional outcomes in refractive surgery and can be improved by increasing the regularity of the ablated surface through final smoothing [37]. Finally, the decentration ablation may have little effect on the visual quality under bright light, whereas in dark light, with pupil dilatation, light passes through the connecting part of the optical zone and the transition zone, resulting in a significant increase in HOAs.

Our study has limitations that should be considered when interpreting the results. First, this study found a relationship between pupil offset and HOAs after refractive surgery. More studies on the relationship between HOAs and vision quality needed. Using visual quality questionnaire assessing subjective visual discomfort may be very important. Second, the patients included in the study were followed up for 3 months, which may not account for the long-term effects of surgery. Further studies with larger sample sizes and different methods to investigate the relationship between pupil offset, HOAs, and visual quality is desirable.

## Conclusions

Our study found that postoperative HOAs and ΔHOAs were associated with preoperative MRSE and pupil offset. The HOAs became more obvious as the MRSE increased in patients with a preoperative pupil offset ≤ 0.20 mm. The increase in postoperative HOAs was more obvious in patients with preoperative pupil offset > 0.20 mm. Based on this change characteristic, pupil offset can be adjusted in the preoperative design of refractive surgery to reduce HOA and improve visual quality. This study has clinical implications because it confirms the significance of excimer laser ablation center position and may provide guidance for achieving accurate refractive error correction results.

## Data Availability

All data and materials were unpublished. For inquiries, please contact the corresponding author directly.

## Abbreviations

CCT: Central corneal thickness
CDVA: Corrected distance visual acuity
FS-LASIK: Femtosecond laser in situ keratomileusis
HOAs: Higher-order aberrations
MRSE: Manifest refraction spherical equivalent
RMS: Root mean square
UDVA: Uncorrected distance visual acuity
